# Successful surgical management of CIC-DUX4 sarcoma of the chest wall

**DOI:** 10.1093/jscr/rjag510

**Published:** 2026-06-26

**Authors:** Forrest Bohler, Meghan Chaiyasate, Joshua R Waggener

**Affiliations:** Oakland University William Beaumont School of Medicine, 586 Pioneer Dr., Rochester, MI 48309, United States; University of Michigan College of Literature, Science, and the Arts, 500 South State Street, Ann Arbor, MI 48109, United States; Department of Surgery, Bitterroot Health - Daly Hospital, 1200 Westwood Dr., Hamilton, MT, 59840, United States

**Keywords:** CIC-DUX4 sarcoma, chest wall tumour, soft tissue sarcoma, surgical excision, rural surgery

## Abstract

CIC-DUX4 sarcoma is a rare and aggressive undifferentiated small round cell sarcoma most commonly affecting young adults and typically managed with multimodal therapy including surgery and systemic chemotherapy. We report a case of a 40-year-old female who presented with a rapidly enlarging fungating mass of the left anterior chest wall. Imaging demonstrated a localized soft-tissue lesion without evidence of metastatic disease. The patient underwent wide local excision of the mass with en bloc resection of involved fascia at a rural critical access hospital. Pathology confirmed CIC-DUX4 sarcoma of the chest wall. Postoperative recovery was uneventful and the patient underwent close oncologic surveillance. Follow-up imaging at two years demonstrated no evidence of recurrence or metastasis. This case highlights the potential role of definitive surgical management for localized CIC-DUX4 sarcoma and demonstrates that complex oncologic surgery can be successfully performed in resource-limited rural settings.

## Introduction

Capicua transcriptional repressor (CIC) rearranged sarcomas are a recently characterized group of undifferentiated small round cell sarcomas that are genetically distinct from Ewing sarcoma and are associated with aggressive clinical behaviour and poor prognosis [1–3]. Among these tumors, CIC-DUX4 fusion sarcomas are the most common subtype and typically arise in the soft tissues of the trunk and extremities in young adults [1, 4]. Compared with Ewing sarcoma, these tumors often demonstrate relative resistance to conventional chemotherapy and are associated with higher rates of recurrence and metastasis.

Standard management strategies generally involve multimodal therapy consisting of surgical resection combined with systemic chemotherapy and, in some cases, radiation therapy [4–6]. However, optimal treatment strategies remain poorly defined because of the rarity of this malignancy and the limited number of reported cases.

We report a case of localized CIC-DUX4 sarcoma of the chest wall presenting as a fungating soft tissue mass that was successfully treated with surgical excision alone at a rural critical access hospital, with no evidence of recurrence on follow-up.

## Case report

A 40-year-old female presented to the emergency department at a critical access hospital in rural Montana with a 6-week history of a rapidly enlarging mass on the left anterior chest wall associated with drainage, intermittent bleeding, and malodor. The patient reported progressive enlargement of the lesion over several weeks with increasing discomfort. She denied systemic symptoms including fevers, chills, weight loss, or night sweats.

Given the patient’s residence in a rural community, referral to the nearest tertiary center would have required travel of <1 hour for evaluation, treatment, and ongoing surveillance. Socioeconomic considerations and the patient’s preference for receiving care locally contributed to the decision to pursue management at her community’s local rural critical access hospital.

On physical examination, a large fungating mass was noted on the left lower anterior chest wall (Fig. 1). The lesion measured ~6 × 6 × 4 cm with a base diameter of ~4 cm and had an ulcerated appearance with areas of necrotic tissue. Given the pigmented and fungating nature of the lesion, melanoma was initially considered in the differential diagnosis.

**Figure 1:**
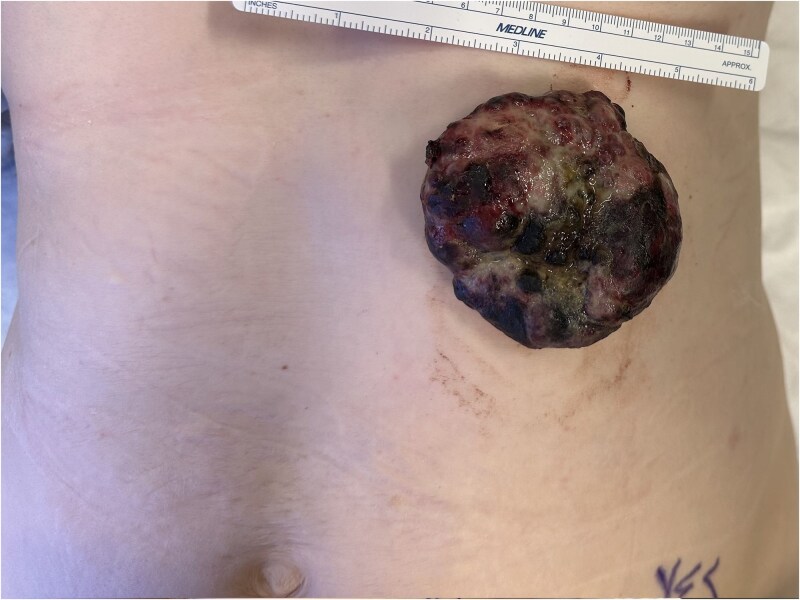
Clinical photograph demonstrating a large fungating lesion measuring ~6 × 6 × 4 cm arising from the left anterior chest wall.

Computed tomography (CT) of the chest, abdomen, and pelvis demonstrated a localized soft tissue mass involving the left anterior chest wall without evidence of metastatic disease. There was no involvement of the underlying thoracic cavity or evidence of distant metastases (Figs 2 and 3).

**Figure 2:**
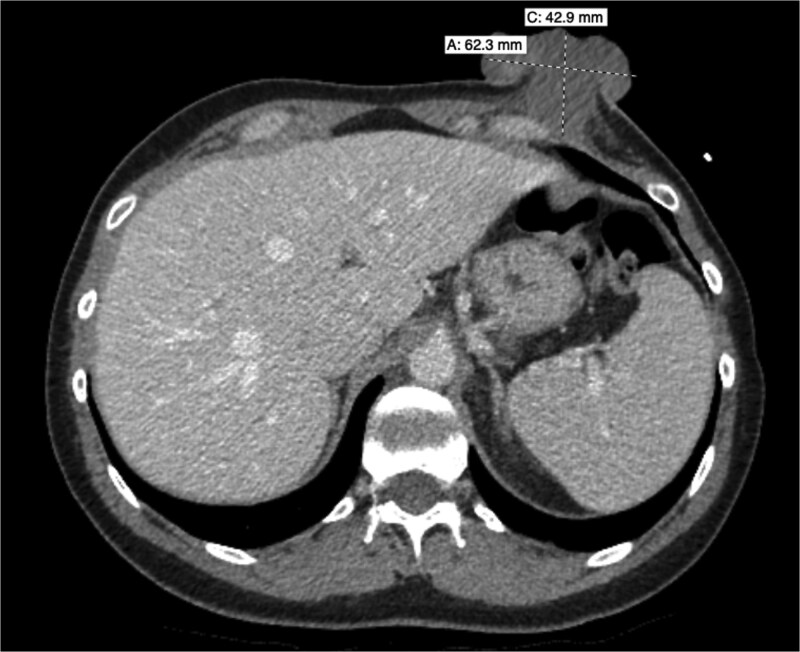
Axial contrast-enhanced CT of the chest demonstrating an exophytic soft tissue mass arising from the left anterior chest wall measuring ~62.3 mm in maximal transverse dimension and 42.9 mm in craniocaudal dimension, extending outward from the subcutaneous tissues without clear invasion of the underlying thoracic cavity.

**Figure 3:**
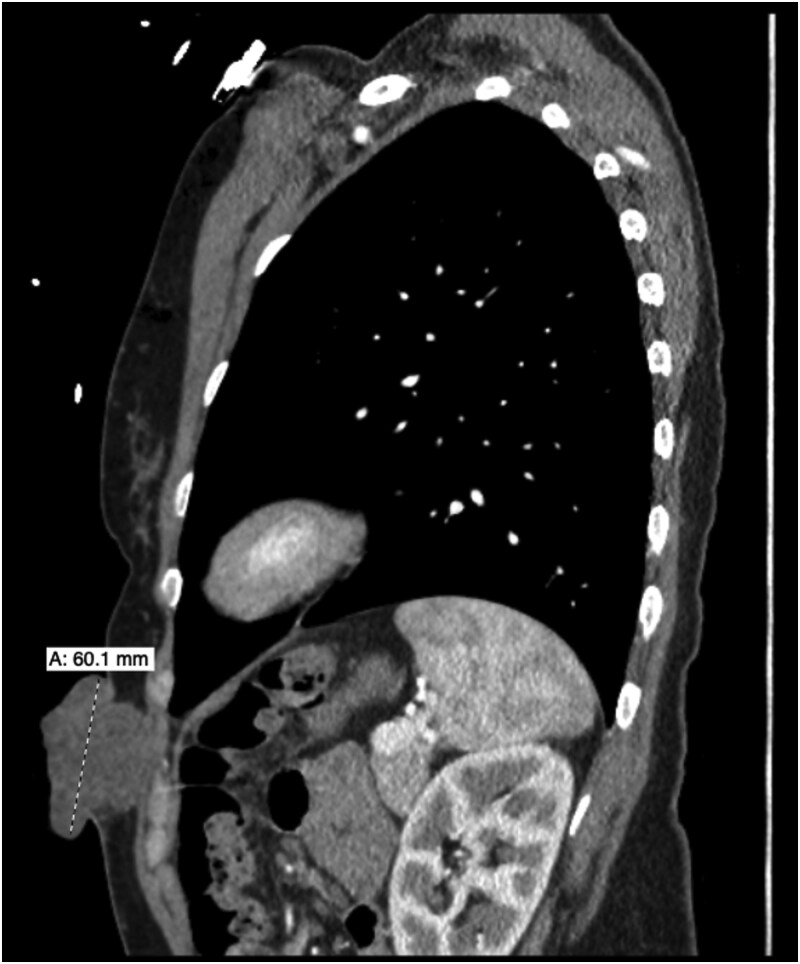
Sagittal contrast-enhanced CT of the chest demonstrating an exophytic soft tissue mass arising from the left anterior chest wall measuring ~60.1 mm in maximal dimension, extending outward from the subcutaneous tissues without clear invasion of the underlying thoracic cavity.

Given the symptomatic nature of the lesion and concern for malignancy, the patient was taken to the operating room for excisional biopsy. The procedure was performed under monitored anesthesia care. A wide transverse elliptical incision was planned to achieve adequate margins around the lesion. An ~18 × 7 cm segment of skin and subcutaneous tissue containing the fungating mass was excised (Fig. 4).

**Figure 4:**
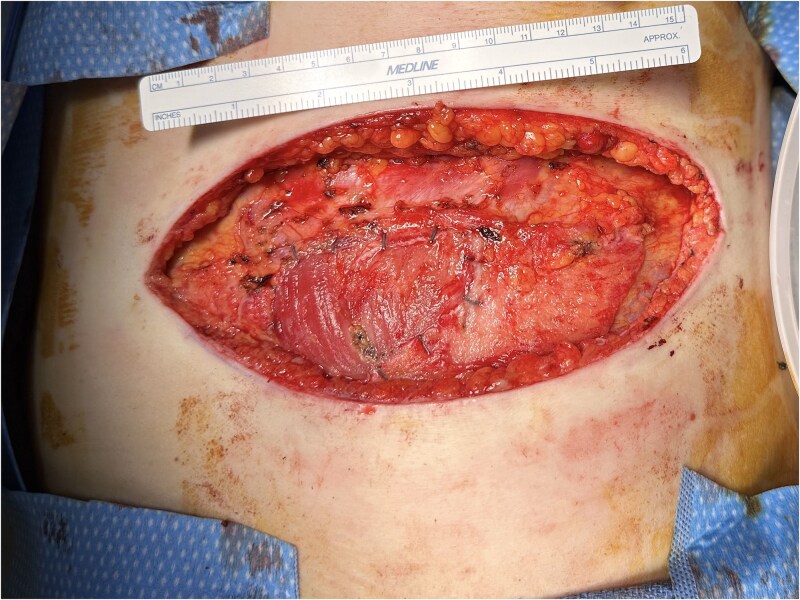
Intraoperative photograph demonstrating the surgical defect following wide local excision of the left anterior chest wall mass. The elliptical incision measures ~18 cm in maximal length, with exposure of the underlying anterior rectus fascia, portions of which were resected en bloc with the tumour to achieve adequate margins.

During dissection, the tumour was found to extend through the subcutaneous tissue and was adherent to the anterior rectus fascia near its insertion onto the ribs. A 4 × 4 cm segment of involved fascia was resected en bloc with the tumor to achieve clear margins. There was no gross invasion into the underlying musculature or thoracic cavity. Haemostasis was obtained and the wound was closed primarily (Fig. 5).

**Figure 5:**
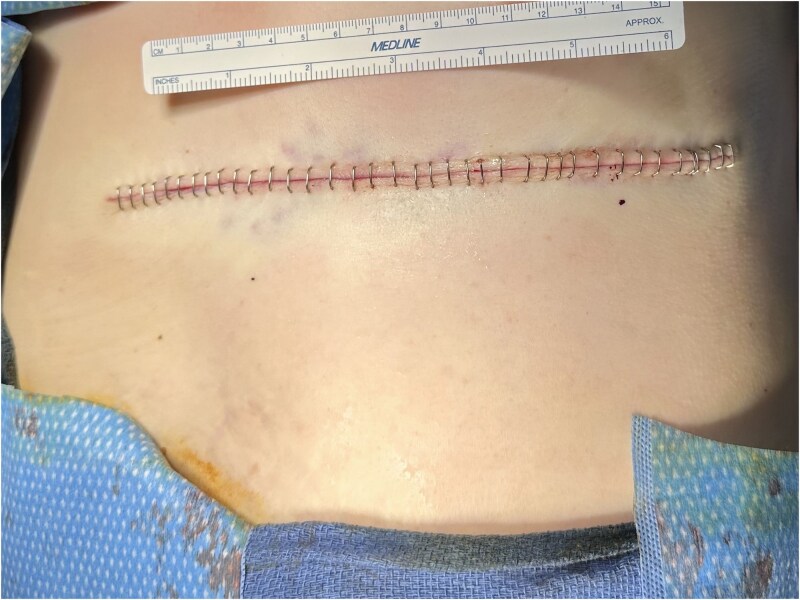
Intraoperative photograph demonstrating primary closure of the surgical defect following wide local excision of the left anterior chest wall mass. The incision measures ~18 cm in maximal length.

The patient tolerated the procedure well and her postoperative course was uncomplicated. She was discharged home in stable condition with close surgical follow-up.

Histopathologic evaluation demonstrated an undifferentiated small round cell sarcoma. Subsequent molecular testing confirmed a CIC-DUX4 rearranged sarcoma of the chest wall.

Following diagnosis, the patient was referred for oncology evaluation. Given the rarity of CIC-rearranged sarcoma and the lack of clear treatment guidelines, the potential role of adjuvant chemotherapy was considered. However, because the tumor had been completely excised with negative margins and staging imaging demonstrated no evidence of metastatic disease, a multidisciplinary decision was made to pursue close surveillance rather than immediate systemic therapy.

The patient underwent regular follow-up with serial imaging. At 2 years following surgical resection, repeat CT imaging of the chest and abdomen demonstrated no evidence of local recurrence or distant metastasis.

## Discussion

This case highlights several important considerations for surgical oncologists. First, CIC-rearranged sarcomas may present with atypical clinical features that can mimic other malignancies. In this patient, the rapidly enlarging fungating lesion initially raised concern for melanoma because of its ulcerated appearance and cutaneous involvement. This diagnostic uncertainty illustrates the importance of maintaining a broad differential diagnosis when evaluating large or rapidly progressing chest wall lesions. In such cases, early surgical excision may serve both diagnostic and therapeutic purposes.

Second, this case demonstrates that complex oncologic procedures can be successfully performed in rural and resource-limited environments. Patients in rural regions frequently face substantial barriers to accessing specialized oncologic care, including long travel distances and delays associated with referral to tertiary centers [7, 8]. In such settings, the presence of a broadly trained general surgeon capable of managing a wide spectrum of surgical pathologies can be critical [9]. Rapidly enlarging or aggressive tumors may progress significantly during prolonged referral and treatment intervals, potentially worsening patient outcomes. In this case, timely surgical intervention at a rural critical access hospital allowed for prompt resection of the tumor and definitive diagnosis without the delays that may have accompanied referral to a distant tertiary center.

Perhaps most important, this case underscores the potential for aggressive surgical resection in the management of localized CIC-DUX4 sarcoma. Although multimodal treatment strategies incorporating chemotherapy and radiation are frequently utilized, the optimal therapeutic approach for CIC-rearranged sarcomas remains poorly defined because of their rarity and the limited effectiveness of conventional Ewing sarcoma–based regimens reported in several studies. Prior literature has suggested that CIC-DUX4 sarcomas may demonstrate relative resistance to systemic therapy and are associated with high rates of recurrence and metastasis despite aggressive multimodal management [1, 5].

In this context, achieving complete surgical excision with negative margins may represent the most important determinant of durable local disease control in select patients with localized disease. In the present case, en bloc resection of the tumour with involved anterior rectus fascia achieved an R0 resection without evidence of residual local invasion into the thoracic cavity or underlying musculature. The patient remains disease-free 2 years following surgery without adjuvant chemotherapy or radiation therapy. Although longer follow-up and additional studies are necessary, this outcome suggests that definitive surgical management alone may provide meaningful long-term disease control in carefully selected patients when complete oncologic resection is feasible.

As recognition of CIC-rearranged sarcomas continues to increase, additional case reports and clinical series will be essential to better define the role of surgery, systemic therapy, and multidisciplinary management strategies. Cases such as this contribute valuable insight into the clinical behavior and treatment outcomes of this rare malignancy and may help inform future treatment approaches.

## References

[1] Antonescu CR, Owosho AA, Zhang L et al. Sarcomas with CIC-rearrangements are a distinct pathologic entity with aggressive outcome: a clinicopathologic and molecular study of 115 cases. Am J Surg Pathol 2017;41:941–9. 10.1097/PAS.0000000000000846 28346326 PMC5468475

[2] Rekhi B, Rumdee R, Shetty O. Clinicopathological features of five cases of *CIC::DUX4* positive sarcomas, including literature review. Ann Diagn Pathol 2023;65:152153. 10.1016/j.anndiagpath.2023.152153 37167753

[3] Brahmi M, Vanacker H, Macagno N et al. CIC-DUX4 sarcomas. Curr Opin Oncol 2022;34:342–7. 10.1097/CCO.0000000000000855 35730520

[4] Maaita W, Hasasna N, Yaser S et al. CIC-rearranged sarcoma: a clinical and pathological study of a peculiar entity. Diagnostics 2025;15:1758. 10.3390/diagnostics15141758 40722508 PMC12293365

[5] Murphy J, Resch EE, Leland C et al. Clinical outcomes of patients with CIC-rearranged sarcoma: a single institution retrospective analysis. J Cancer Res Clin Oncol 2024;150:112. 10.1007/s00432-024-05631-7 38436779 PMC10912249

[6] Ponce RKM, Luck C, Okimoto RA. Molecular and therapeutic advancements in Capicua (CIC)-rearranged sarcoma. Front Cell Dev Biol 2024;12:1416697. 10.3389/fcell.2024.1416697 38882060 PMC11176417

[7] Charlton M, Schlichting J, Chioreso C et al. Challenges of rural cancer care in the United States. Oncology 2015;29:633–40. 26384798

[8] Onega T, Duell EJ, Shi X et al. Geographic access to cancer care in the U.S. Cancer 2008;112:909–18. 10.1002/cncr.23229 18189295

[9] Heneghan SJ, Bordley J, Dietz PA et al. Comparison of urban and rural general surgeons: motivations for practice location, practice patterns, and education requirements. J Am Coll Surg 2005;201:732–6. 10.1016/j.jamcollsurg.2005.06.262 16256916

